# Two-Dimensional Quantum Genetic Algorithm: Application to Task Allocation Problem

**DOI:** 10.3390/s21041251

**Published:** 2021-02-10

**Authors:** Sabyasachi Mondal, Antonios Tsourdos

**Affiliations:** School of Aerospace, Transport and Manufacturing (SATM), Cranfield University, Cranfield MK430AL, UK; a.tsourdos@cranfield.ac.uk

**Keywords:** Quantum Genetic Algorithm, two-dimensional quantum chromosome, task allocation

## Abstract

This paper presents a Two-Dimensional Quantum Genetic Algorithm (2D-QGA), which is a new variety of QGA. This variety will allow the user to take the advantages of quantum computation while solving the problems which are suitable for two-dimensional (2D) representation or can be represented in tabular form. The performance of 2D-QGA is compared to two-dimensional GA (2D-GA), which is used to solve two-dimensional problems as well. The comparison study is performed by applying both the algorithm to the task allocation problem. The performance of 2D-QGA is better than 2D-GA while comparing execution time, convergence iteration, minimum cost generated, and population size.

## 1. Introduction

The Genetic algorithm (GA) [1] is a class of bio-inspired algorithms that can produce the optimal or near-optimal solution of complex optimization problems in a reasonable time. It was first proposed by Holland [2] inspired by Darwin’s principle of survival of the fittest. The possible solution of an optimization problem is encoded in a chromosome which consists of an array of bits called genes. The individual chromosome is evaluated by a fitness function. A genetic population consists of a finite number of chromosomes. The chromosomes of the new population are generated by the application of genetic operations such as crossover, mutation, and reproduction on the present population. The new population optimizes the fitness function and thus provides an improved solution. The solution thus approaches the optimal solution over several generations. There exist many applications of GA viz. optimization [3,4], machine learning [5], neural networks [6], fuzzy logic controllers [7], identification [8], fault diagnosis [9], and financial market [10].

As quantum technology emerges as a powerful computational tool, there was an effort to combine quantum computation [11,12] with intelligent optimization algorithms. The first attempt to combine quantum mechanics principles and Genetic Algorithm was made by Narayanan and Moore to propose Quantum Genetic Algorithm (QGA) [13]. QGA is applicable to the category of problems which are solved using conventional genetic algorithm. Moreover, QGA speeds up the computation of genetic evolutionary process by exploiting the power of quantum computation. QGA has higher convergence rate, less execution time, less population size, and strong global search capability [14,15,16]. QGA has been proved to be efficient in solving various kind of problems such as combinatorial and functional optimization problems, engineering optimization problems, image processing and identification, and many others. A few example application can be found in [14,17,18,19,20,21,22,23,24] and many more.

The GA and QGA discussed so far consider the chromosomes as a one-dimensional array. Obviously, these chromosomes cannot represent a possible solution for the problems which naturally have two-dimensional representation. Examples of such problems include Ising problem, Packing problem, Scheduling problem, Optimal topology in Multi-Agent Systems etc. These type of problems were solved using 2D-GA. Ising problem is discussed in [25]. Ising model is a famous model which is used to study thermodynamic properties, magnetic spin correlations, phase transitions, and many other applications. In general, the problem is to minimize the total energy function for a given matrix *E* of interaction energies and external field *H* by finding an assignment of the spin variables. Packing problem is discussed in [26]. The primary purpose of this kind of problems is to obtain a high packing density in two or three dimensions. Examples of such problem are the layout of mechanical and electromechanical components and assemblies, bin packing, and container and car loading. Industries such as transportation, glass, leather Packing etc. require packing as well. 2D-GA can solve the 2D layout or packing problem successfully. In [27], 2D-GA is implemented to address the scheduling problem. A variety of two-dimensional crossover and mutation operations are presented to generate a new population. The proposed algorithm was applied to solve the aircraft scheduling problem. In [28], a genetic algorithm with a multidimensional chromosome is used to describe nodes belonging to multiple communities in dense overlapping communities. Another critical and new application of 2D-GA is found in [29,30]. The objective of this paper is to obtain an optimal communication topology to achieve consensus among agents such that the consensus control energy of the agents is minimized.

Although 2D-GA solves the problems with 2D representation, it does not inherit the computation power such as QGA. Keeping this in mind, the two-dimensional-QGA (2D-QGA) is proposed in this paper to solve the 2D problems. The genetic evolutionary processes in 2D-QGA inherit the power of quantum computation to solve the 2D problems with higher convergence rate, less execution time, and less population size. In the algorithm, the two-dimensional quantum population is generated, and two new Qgate rotation processes (named ‘type-I’ and ‘type-II’) are proposed to generate the new population for the next generation. The proposed algorithm is applied to solve the Multi-robot task allocation problem, which can be represented in two dimensions. The 2D-QGA and 2D-GA are compared based on execution time, iteration, and population size. Performance of 2D-QGA is found to be better compared to 2D-GA.

The contributions of this work are given below:

1. A novel 2D-QGA is proposed in this paper, which is a new algorithm in GA family. Thus, the power of quantum computation can be used to solve problems having two-dimensional representation. The quantum computation speeds up the computation and delivers optimal results in less time with a smaller population size.
2. New Qgate rotation technique for 2D chromosomes is proposed in this work. This Qgate is the key factor to speed up the computation in 2D-QGA.
3. The proposed algorithm is implemented to multi-robot task allocation problem, which has the two-dimensional form and it is egarded as NP-hard.
4. A detailed analysis of the convergence time, iteration, cost, execution time of 2D-QGA is discussed. It also includes a detailed comparison with existing 2D-GA.

The rest of the paper is organized as follows. In Section 2, details of QGA is presented, which is the preliminary for two-dimensional QGA. In Section 3, 2D-QGA is introduced. An application of the algorithm is shown in Section 4. The comparison study is presented in Section 5. A brief conclusion is given in Section 6.

## 2. Quantum Genetic Algorithm (QGA)

In this section, the Quantum Genetic Algorithm is discussed, which is the preliminary for two-dimensional QGA. In quantum computation, $2^{m}$ independent states can be represented using *m* qubits. However, during the measurement of the qubits, a single state of the quantum state (i.e., |0〉 or |1〉) is obtained, which is similar to a conventional genetic algorithm, i.e., a chromosome’s representation is defined as a string of *m* information units.

### 2.1. Qubit Representation

Qubit is the basic unit of information used in quantum computations. In a quantum system, the basic state is not deterministic; i.e., it does not have a fixed value. The basic state maybe |0〉, |1〉, or any complex value which is a linear combination or superposition of basis states. A qubit is represented in Equation (1) as

(1) |q〉=α|0〉+β|1〉

where α and β are complex numbers which signify the magnitude of left and right vector respectively. α and β satisfy the condition given in Equation (2).

(2) $${\left|\alpha \right|}^{2}+{\left|\beta \right|}^{2}=1$$

**Figure 1:**
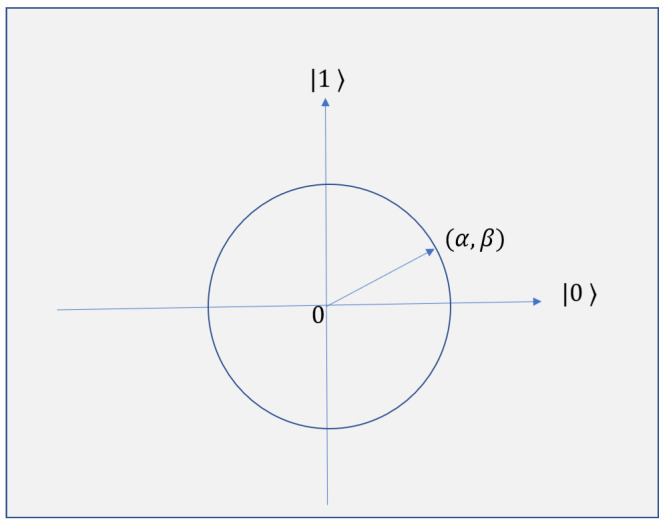
Qubit representation.

${\left|\alpha \right|}^{2}$ and ${\left|\beta \right|}^{2}$ provide the probabilities that the qubit can be found in the ‘0’ state and ‘1’ state, respectively. The pictorial representation of qubit is given in Figure 1.

A chromosome is represented by a string of qubits (given in Equation (3)). The encoding length of the chromosome is *n*.

(3) $$\left[\begin{array}{c} \alpha_{1}\\ \beta_{1} \end{array}|\begin{array}{c} \alpha_{2}\\ \beta_{2} \end{array}|\ldots |\begin{array}{c} \alpha_{n}\\ \beta_{n} \end{array}\right]$$

where $|\alpha_{i}{|}^{2}+{\left|\beta_{i}\right|}^{2}=1,i=1,2,\ldots ,n$. The genes are represented by each pair of $\left(\alpha_{i},\beta_{i}\right)$, i=1,2,…,n. In the next section Qgate rotation which is used to obtain new generation in QGA is discussed.

### 2.2. Quantum Gate Rotation (Qgate)

The operation for changing the state of the qubit is known as the quantum gate or Qgate. There exist several quantum gates. Examples of such gates are Not gate, controlled-NOT gate, rotation gate, Hadamard gate etc. One of the widely used gates is the rotation gate which is described as follows.

The rotation gate is operated on the present population to obtain the new one. It helps to maintain the diversity of the population, which is a key updating operation in quantum evolution algorithm. Rotation gate $U(\Delta \theta_{i})$ is employed to update the probability amplitude of quantum states. The *i*th qubit $\left(\alpha_{i},\beta_{i}\right)$ is updated as follows.

(4) $$\left[\begin{array}{c} {\bar{\alpha }}_{i}\\ {\bar{\beta }}_{i} \end{array}\right]=T\left(\theta_{i}\right)\left[\begin{array}{c} \alpha_{i}\\ \beta_{i} \end{array}\right]$$

where

$$T\left(\theta_{i}\right)=\left[\begin{array}{cc} \cos(\theta_{i}) & -\sin(\theta_{i})\\ \sin(\theta_{i}) & \cos(\theta_{i}) \end{array}\right]$$

where *T* is a rotation matrix and $\theta_{i}=s\left(\alpha_{i},\beta_{i}\right)\Delta \theta_{i}$ is rotation angle. $s(\alpha_{i},\beta_{i})$ is the sign of $\theta_{i}$ and it determines the rotation direction. $\Delta \theta_{i}$ is the amount of rotation. The rotation sign and magnitude is determined in a look-up table which is shown in Table 1.

**Figure 2:**
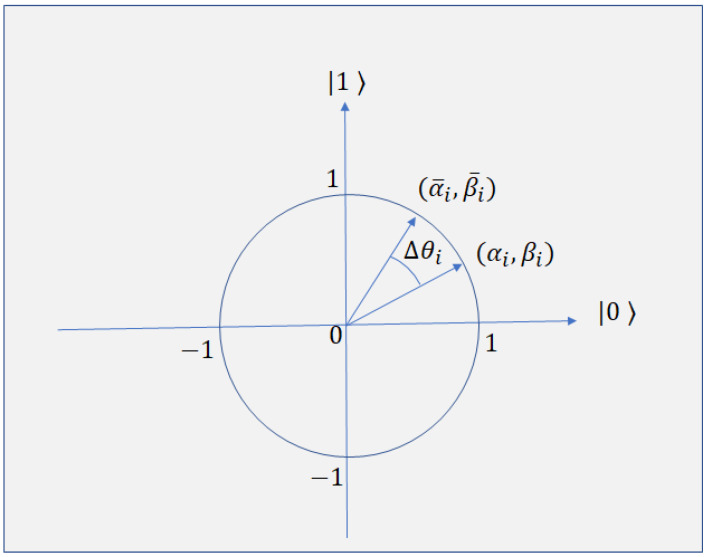
Rotation gate for QGA.

The rotation gate for qubit individuals in a polar plot is shown in Figure 2. The solution $\left[\begin{array}{c} \alpha_{i}\\ \beta_{i} \end{array}\right]$ of current generation is rotated by an angle of $\Delta \theta_{i}$ anticlockwise to obtain possible best solution $\left[\begin{array}{c} {\bar{\alpha }}_{i}\\ {\bar{\beta }}_{i} \end{array}\right]$.

$r_{i}$ corresponds to the binary code of individual chromosome *i*. The binary code corresponding to the best individual is $b_{i}$ and f(.) denotes the fitness function to find the fitness value. $\alpha_{i}\beta_{i}>0$ signifies that they lie in the first or third quadrant. If $\alpha_{i}\beta_{i}<0$ then the individual *i* lies in the second or fourth quadrant. If the combination is 0-1, set the rotation angle to be 0.5π. If the combination is similar, i.e., 0-0 and 1-1, select a smaller rotation of angle 0.2π. If it is not possible to determine specific quadrant, then rotation is not preferred.

### 2.3. Initial Population Generation

The qubits present in the initial population have equal probability to be in the state of |0〉 and |1〉. For such case, $\alpha =\frac{1}{\sqrt{2}}$ and $\beta =\frac{1}{\sqrt{2}}$, i.e., $|q\rangle =\frac{1}{\sqrt{2}}|0\rangle +\frac{1}{\sqrt{2}}|1\rangle$. The chromosomes have string of such α and β. The encoding length of the chromosome is *n* and the population size is *N*. The algorithm for generation population is given in Algorithm 1.
**Algorithm 1** Initial population of quantum chromosomes Init_Pop_QGA() **for**
i=1 to *N*
**do**  **for**
j=1 to *n*
**do**   $C\left(1,j\right)\leftarrow \frac{1}{\sqrt{2}}\;\%\%\%\;$ for α   $C\left(2,j\right)\leftarrow \frac{1}{\sqrt{2}}\;\%\%\%\;$ for β  **end for**  Ch(:,:,i)←C **end for**

### 2.4. Generate Binary String by Measuring Quantum Chromosome

The function for measuring the quantum chromosome measure_chrom() is shown in Algorithm 2. In this algorithm, a random number x∈(0,1) is generated by MATLAB function rand() and it is compared to each $\alpha_{j}^{2}$ (denoted as $Chrom{\left(1,j\right)}^{2}$) of the chromosome.
**Algorithm 2** Initial population of quantum chromosome measure_chrom() **for**
k=1 to *N*
**do**  Chrom←Ch(:,:,k)  **for**
j=1 to *n*
**do**   x←rand   **if**
$x>{\left(Chrom\left(1,j\right)\right)}^{2}$
**then**    B(j)←0   **else**    B(j)←1   **end if**  **end for**  binary(:,:,k)←B **end for**

If $x>\alpha_{ij}^{2}$ the the (i,j)th element of a temporary matrix *B* is assigned as 0; otherwise assigned as 1. This matrix *B* is stacked in a 3D matrix binary.

### 2.5. QGA Algorithm

For better understanding, the QGA algorithm is shown in Algorithm 3. It includes the operations for QGA which are described so far.
**Algorithm 3** QGA algorithm Initialize QGA parameters Generate initial quantum population Q(t) **for**
k=1 to *N*
**do**  Generate binary initial population P(t)  Store the best solution *b* among P(t)  **if** converged **then**   Stop  **else**   Obtain Q(t+1) by updating Q(t) using Qgate   Set t←t+1  **end if** **end for**

## 3. Two-Dimensional QGA (2D-QGA)

Two-dimensional QGA is a new algorithm which inherits the properties of QGA, and applicable to problems which can be represented by two-dimensional chromosome. The major works are done to present the idea of 2D quantum chromosome and its rotation technique. The description of the 2D quantum chromosome and Qgate rotation is given in the following sections.

### 3.1. Two-Dimensional Quantum Chromosome

**Figure 3:**
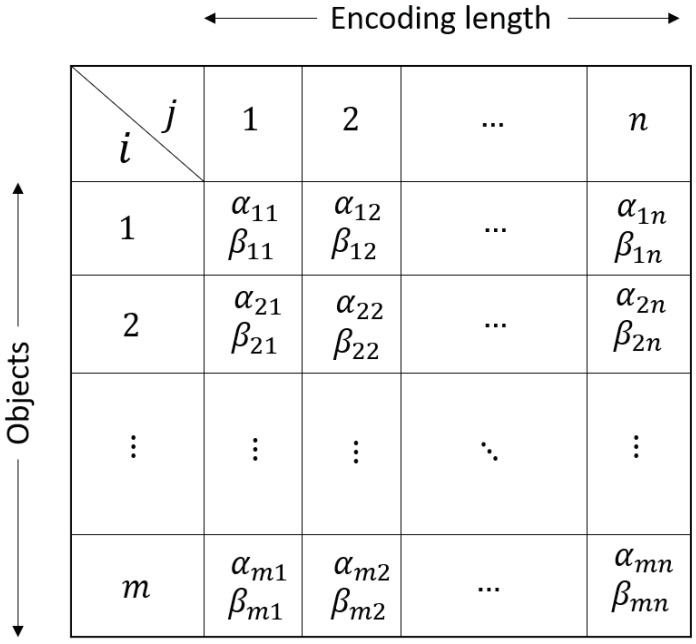
Two-dimensional quantum chromosome representation. Each row is a string of qubits of length *n*.

In contrast to QGA, a two-dimensional quantum chromosome is a matrix. Each row of the matrix signifies a gene. A diagram of the quantum chromosome is shown in Figure 3. ‘i=1,2,…,m’ denotes the objects, and each of them is encoded by a string of qubits of length *n*. It can be mentioned that the quantum chromosomes encode the instants of all the objects together to represent a solution of a 2D problem. The number of rows is double the number of objects since each row is a string of qubits α and β.

**Figure 4:**
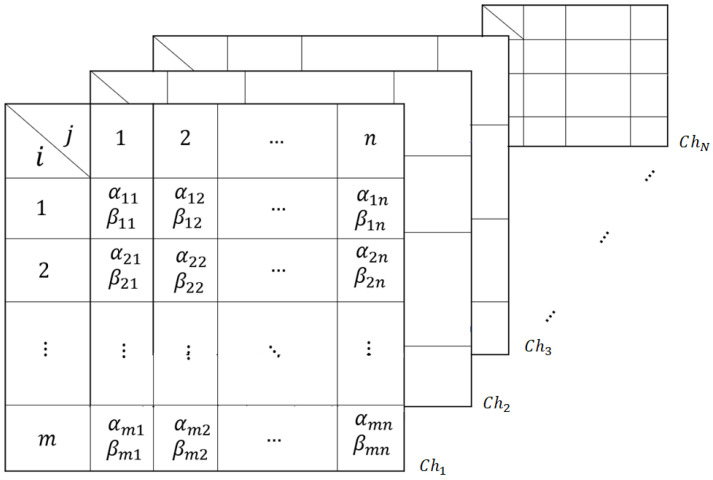
Two-dimensional quantum population with chromosome represented as $Ch_{i,i=1,2,\ldots ,N}$.

The quantum population consist of *N* two-dimensional quantum chromosomes $Ch_{1},Ch_{2},\ldots ,Ch_{N}$ as shown in Figure 4. The algorithm for the generation of the population is given in the following section.

### 3.2. Initial Population Generation

The initial population generation for 2D-QGA is slightly different from QGA. The method Init_Pop_2D() is shown in Algorithm 4.
**Algorithm 4** Initial Population of 2D-Quantum Chromosome Init_Pop_2D() **for**
k=1 to *N*
**do**  **for**
i=1 to *m*
**do**   **for**
j=1 to *n*
**do**    $C\left(2i-1,j\right)\leftarrow \frac{1}{\sqrt{2}}\;\%\%\%\;$ for α    $C\left(2i,j\right)\leftarrow \frac{1}{\sqrt{2}}\;\%\%\%\;$ for β   **end for**  **end for**  Chrom(:,:,k)←C **end for**

**Figure 5:**
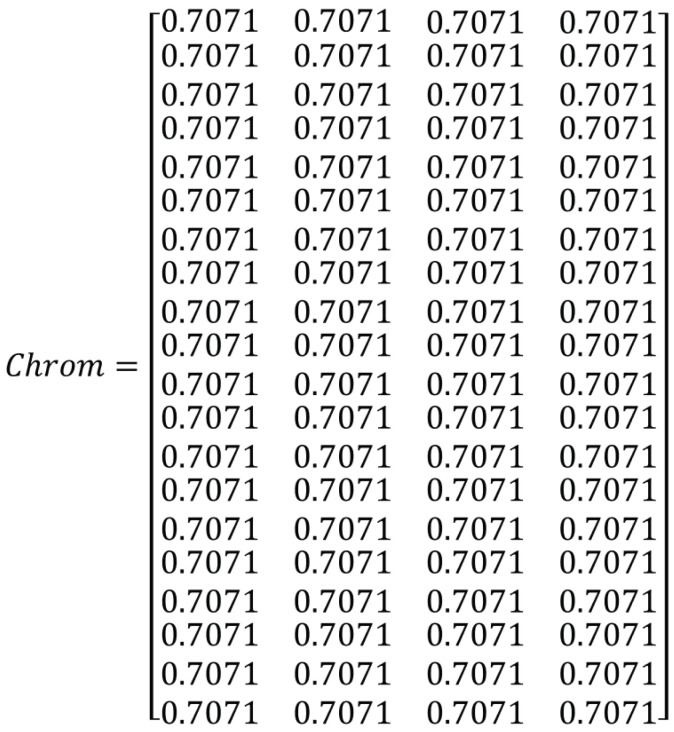
Sample chromosome generated using Algorithm 4.

A common strategy is to initially populate the chromosome such that the qubits appear with equal probability i.e., $\alpha =\beta =\frac{1}{\sqrt{2}}$ and they change as the algorithm is executed. Therefore, the initial values of qubits are assigned as $\frac{1}{\sqrt{2}}=0.7071$. A sample quantum chromosome is shown in Figure 5. This is a chromosome generated using Algorithm 4.

### 3.3. Generation of Binary Population

The binary population is generated from quantum population using Algorithm 5.
**Algorithm 5** Binary population generation from quantum chromosome **for**
k=1 to *N*
**do**  Chrom←Ch(:,:,k)  **for**
i=1 to *m*
**do**   **for**
j=1 to *n*
**do**    x←rand    **if**
$x>{\left(Chrom\left(2i-1,j\right)\right)}^{2}$
**then**     B(i,j)←0    **else**     B(i,j)←1    **end if**   **end for**  **end for**  binary(:,:,k)←B **end for**

**Figure 6:**
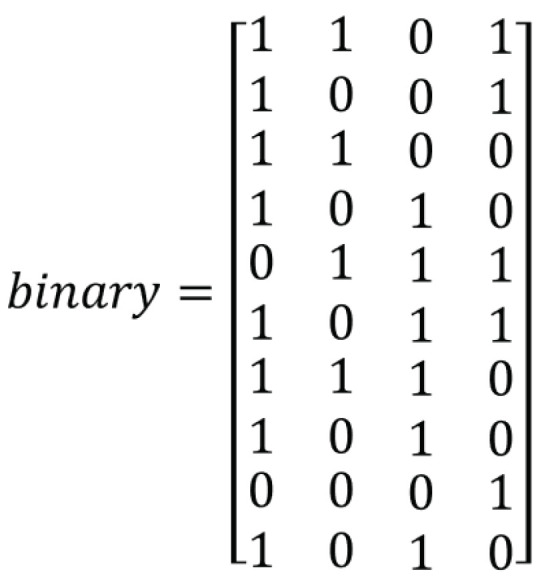
Binary chromosome generated using Algorithm 5.

This algorithm is similar to the binary population generation for QGA. The modification is done to apply it for 2D chromosomes. An example of a binary chromosome generated is shown in Figure 6. It is the binary chromosome generated considering the quantum chromosome given in Figure 5.

### 3.4. Qgate Rotation for Two-Dimensional Chromosome

The rotation for 2D quantum chromosome is different because unlike the QGA (each chromosome dimension 2 × n) the chromosomes of 2D-QGA have the dimension of 2m × n. In this paper, two types of quantum gate rotation technique for two-dimensional quantum chromosome are presented. They are described in the following sections.

#### 3.4.1. Qgate Rotation Type-I

Each 2D quantum chromosome is a matrix, as shown in Figure 3. It can be noted that each row of this chromosome is similar to the chromosome of QGA (i.e.,1D array). In rotation type-I, the Qgate operation for 2D-QGA is performed for each row using the Qgate for QGA (shown in Table 1). The algorithm for implementing Qgate type-I operation for 2D quantum chromosome is shown in Algorithm 6.
**Algorithm 6** Qgate rotation of 2D Quantum Chromosome **for**
i=1 to *N*
**do**  **for**
j=1 to *m*
**do**   Perform checks and operation in Table 1  **end for** **end for**

#### 3.4.2. Qgate Rotation Using N-dimensional Rotation Matrix (Qgate Type-II)

In this type, each column vector of the 2D chromosome is rotated. The length of the column vector is 2m. Therefore the rotation matrix should have the dimension 2m × 2m. Fortunately, there exists a well-defined method for such operation, which is known as ‘Rodrigues’ rotation formula’. The 2D quantum chromosomes are rotated using this method. The definition is given as follows.

**Definition** **1.** *If u and v are two orthonormal vectors, a matrix that rotates the span of u and v by angle θ is*

(5) $$R=I+\sin\theta \left(vu^{T}-uv^{T}\right)+\left(\cos\theta -1\right)\left(uu^{T}+vv^{T}\right)$$

In every generation, each chromosome is rotated by an angle θ, i.e., all columns (${ℜ}^{2m}$) of each 2D quantum chromosome are rotated by the same angle. Rotation angle at each generation is different, and it is selected in a random manner. The sign of the rotation is also random. The rotation process is shown in Algorithm 7.

The population size is *N*. The chromosomes are represented by Ch(:,:,i),i=1,2,…,N. The sign of the rotation i.e., *s* is selected in random manner. In the algorithm, *x* is assigned random number between 0 and 1 by MATLAB function rand(). if x>0.5, then assign s=1; otherwise assign s=−1. The value of θ is obtained by θ=s∗rand()∗(0.1π). Next, a random matrix *M* is generated. Then *M* is converted to orthonormal matrix $M_{orth}$ by MATLAB function orth(). Each rows of *M* are orthogonal. Two rows of *M* (for example, first and second row as shown in the algorithm) are selected as *u* and *v*. The rotation matrix R(θ,u,v) is calculated using values of θ,u, and *v*. Finally, the chromosomes are rotated using the rotation matrix. It can be mentioned that, for each chromosome the values of ‘*s*’ and ‘θ’ are random. Therefore each chromosome is rotated with different rotation angle.

**Figure 7:**
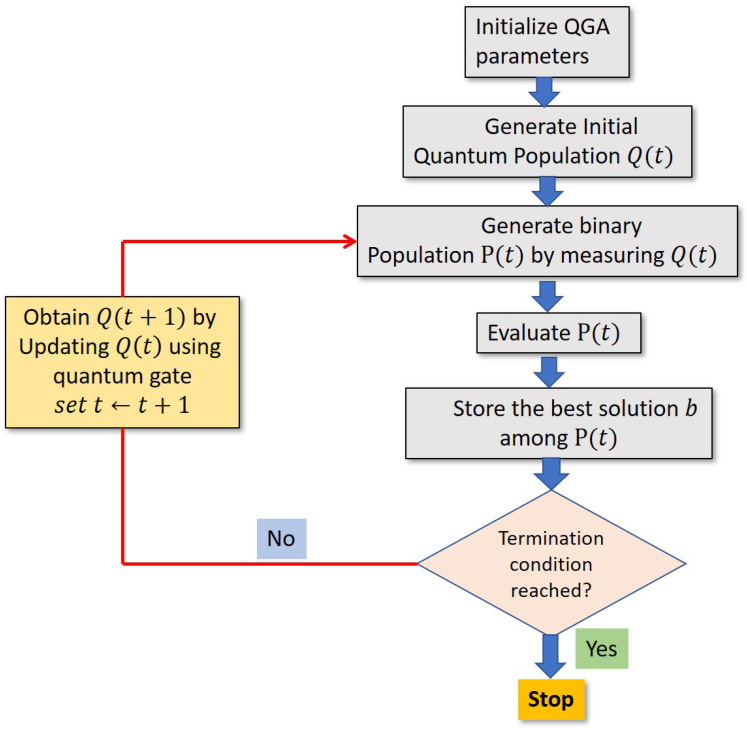
Flow diagram of 2D-QGA.

The flow diagram for implementing the steps of the proposed algorithm is shown in Figure 7.
**Algorithm 7** Qgate Rotation of 2D quantum chromosome **for**
i=1 to *N*
**do**  x←rand()  **if**
x>0.5
**then**   s=1  **else**   s=−1  **end if**  θ←s∗rand()∗(0.1π)  M←rand(2m,2m)  $M_{orth}\leftarrow orth\left(M\right)$  u←M(1,:)  v←M(2,:)  $R\left(\theta ,u,v\right)\leftarrow I+\sin\theta \left(vu^{T}-uv^{T}\right)+\left(\cos\theta -1\right)\left(uu^{T}+vv^{T}\right)$  Ch(:,:,i)←R(θ,u,v)∗Ch(:,:,i) **end for**

## 4. Application to Task Allocation Problem

The problems which naturally have two-dimensional representation can be solved using 2D-QGA, which considers 2D quantum chromosome as a probable solution. It can be mentioned that the task assignment problem can be represented in two-dimension. In this paper, we will study the implementation of 2D-QGA to solve task assignment problem. It is important to note that the problem considered here is for demonstration purpose. The main focus of the work is to show the implementation of the algorithm to such a problem. The assumptions made for this problem are given as follows.

1. Each drone can perform one task at a time i.e., each drone is assigned to one task at a time.
2. At least one drone is required to finish each task
3. All drones should be assigned with a task
4. The task and drone positions are known

The problem is formulated considering these assumptions and given in the following section.

### 4.1. Problem Formulation

The set of task *T* is denoted by $T=[T_{1},T_{2},\ldots ,T_{N_{T}}]$, where $N_{T}$ is the number of tasks. These tasks are considered to be scattered in a field of specific length and width. The set of drones *D* is denoted by $D=[D_{1},D_{2},\ldots ,D_{N_{D}}]$, where number of drones are denoted by $N_{D}$. The number of drones ($N_{D}$) is higher than the number of tasks ($N_{T}$), i.e., $N_{D}>N_{T}$. The allocation problem is given as follows.

(6) $$J=\sum_{i=1}^{N_{D}}\sum_{j=1}^{N_{T}}x_{ij}d_{ij}$$

(7) $$\begin{array}{ccc} \sum_{j}^{N_{T}}x_{ij} & = & 1 \end{array}$$

(8) $$\begin{array}{ccc} \sum_{i}^{N_{D}}x_{ij} & \geq & 1 \end{array}$$

$$x_{ij}=0,1$$

Equations (6)–(8) defines a constrained optimization problem. The constraints in Equations (7) and (8) signifies the assumptions 1–3. The variable $d_{ij}$ is the distance between *i*th drone and *j*th task. $x_{ij}$ is a binary variable which takes a value of 1 if *i*th drone is assigned to *j*th task, i.e., $x_{ij}=1$, otherwise $x_{ij}=0$. This cost function is purely distance-based. The objective of the cost function is to minimize the overall distance between the drones and task positions i.e., assign tasks to the drones depending on their distance from them.

### 4.2. Simulation Study

The simulation study is performed considering ten drones (i.e., $N_{D}=10$; $D=[D_{1}\;D_{2}\;\ldots \;D_{N_{D}}]$) and four tasks (i.e., $N_{T}=4$; $T=[T_{1}\;T_{2}\;T_{3}\;T_{4}]$). It can be mentioned that, this is an example problem. The user can increase the problem size as per the requirement. The task and drones positions are generated in a random manner in an area of length 1000 m and width 100 m using the MATLAB command ‘randi’. The task allocation problem has been solved using both types of 2D-QGA, i.e., type-I and II. The simulation is performed on a PC with AMD Ryzen5 (2.3 GHz) processor and 8 Gb RAM. The results obtained using the proposed algorithm are discussed in the following sections.

#### 4.2.1. Results Obtained Using 2D-QGA with Qgate Type-I

**Figure 8:**
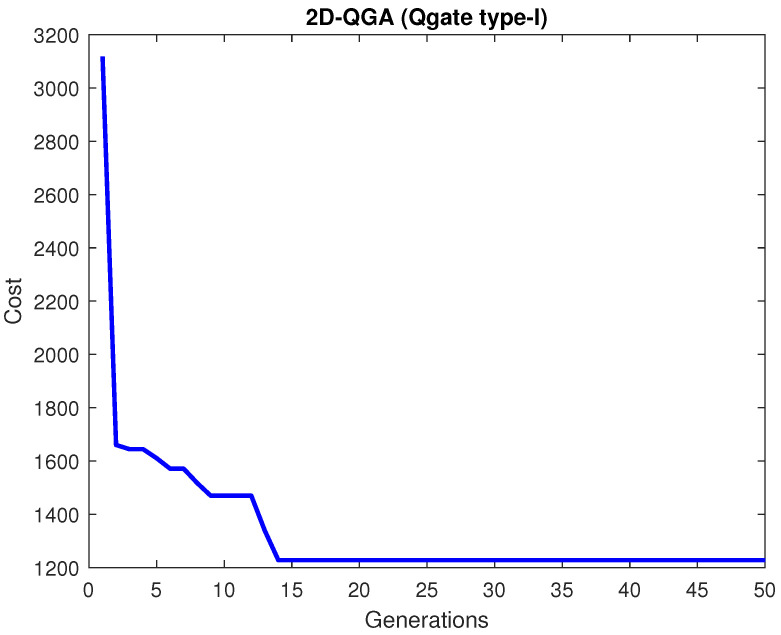
Cost generated by 2D-QGA type-I.

The results generated using 2D-QGA with Qgate type-I is shown here. The algorithm is executed for fifty generations. The cost (*J*) generated for all generations is shown in Figure 8.

**Figure 9:**
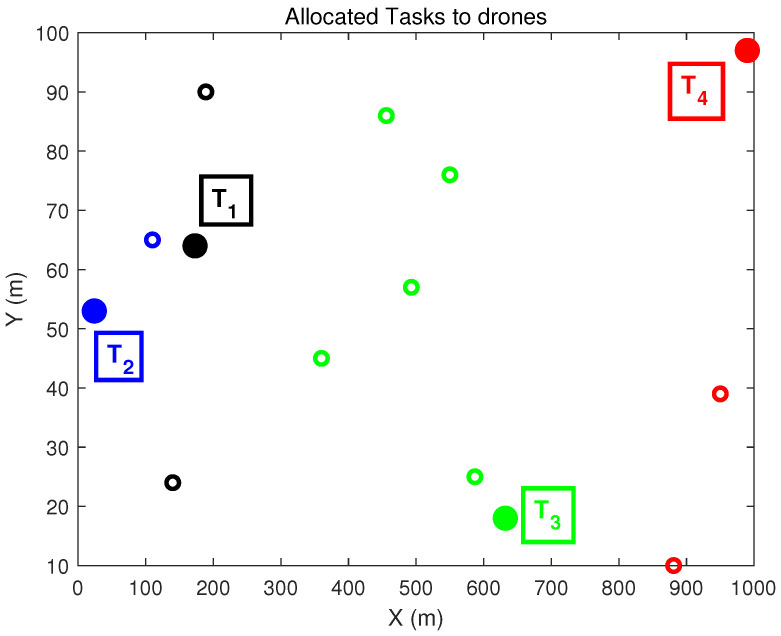
Tasks allocated to the drones (2D-QGA type-I).

The algorithm has converged in fourteen generations to achieve minimum cost. The assignment matrix (optimal solution) is a 2D chromosome. This chromosome shows the optimal assignment of drones to the tasks, as shown in Figure 9. A particular task and the drones assigned to that task are marked with the same colour. The tasks $T=[T_{1}\;T_{2}\;T_{3}\;T_{4}]$ are shown in solid circles, and the drones are shown as empty circles.

**Figure 10:**
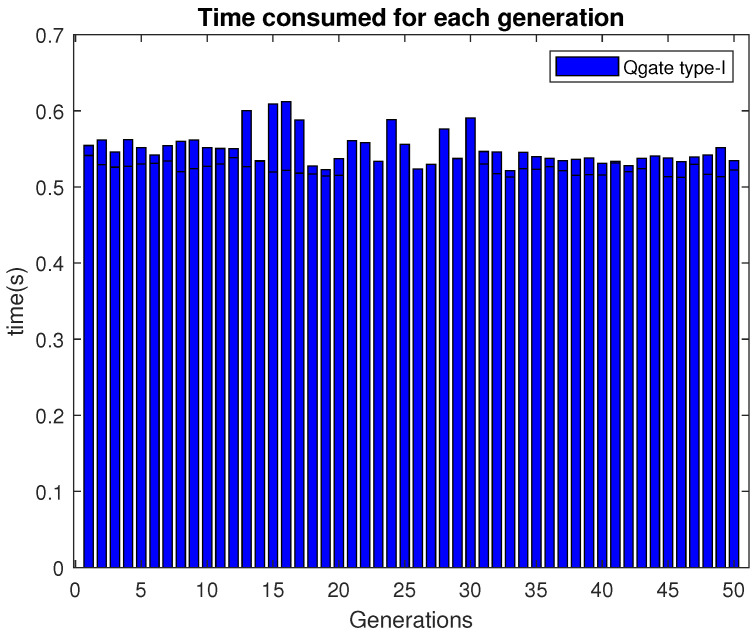
Time consumed per generation by 2D-QGA type-I.

It can be noticed that each drone is assigned to one task. At least one drone is allocated to execute each task. Therefore the constraints are satisfied. The time consumed to execute each generation is shown in Figure 10. It can be observed that most of the generation consumed around 0.57 s.

**Figure 11:**
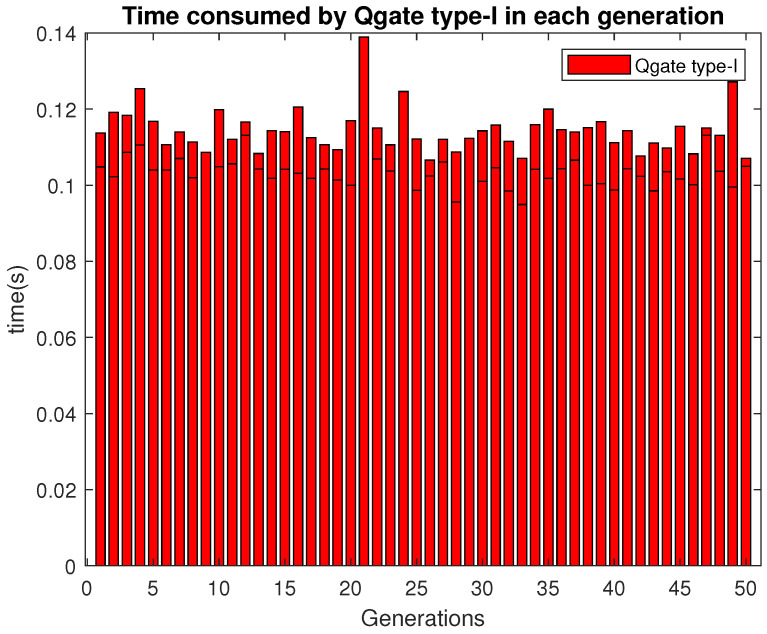
Time consumed by Qgate type-I.

The Qgate type-I consumes around 0.11 s in most of the generations as shown in Figure 11.

#### 4.2.2. Results Obtained Using 2D-QGA with Qgate Type-II

**Figure 12:**
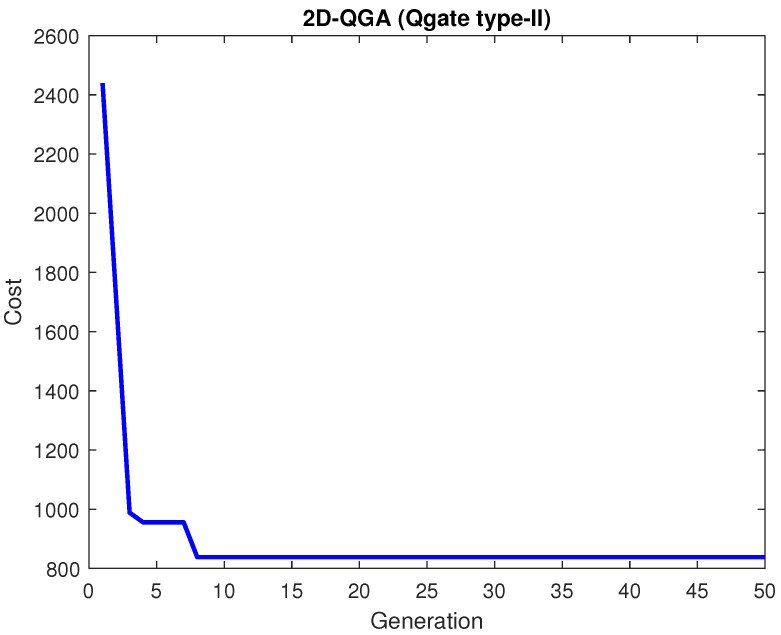
Cost generated by 2D-QGA type-II.

In this case, a different set of task and drone positions are generated. The results are generated using 2D-QGA with Qgate type-II. The cost for all generations is shown in Figure 12. The algorithm converged in 8 generations to produce minimum cost.

**Figure 13:**
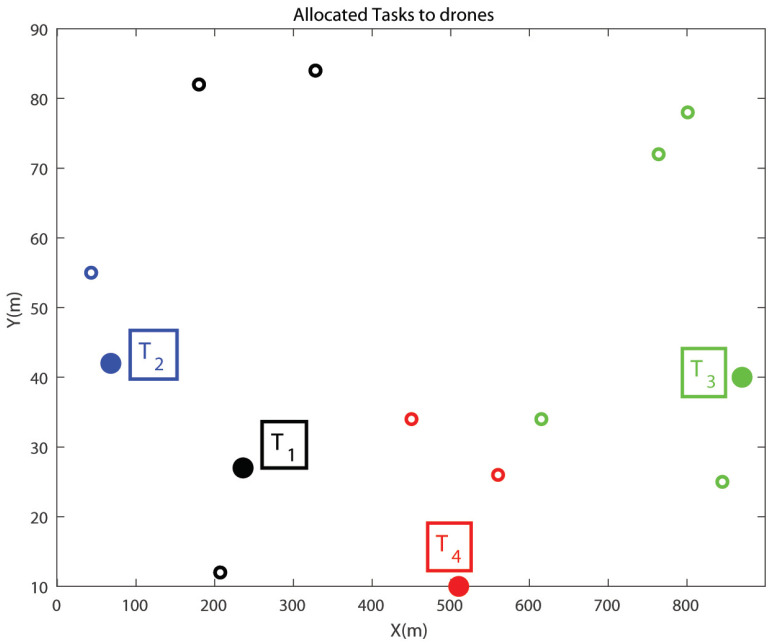
Tasks allocated to the drones (2D-QGA type-II).

The task allocation to the drones is shown in Figure 13. The tasks $T_{1},T_{2},T_{3},T_{4}$ and the drones assigned to the tasks are shown in the same colour as described in the previous case. Also, the tasks are shown in solid circles and drones in empty circles.

**Figure 14:**
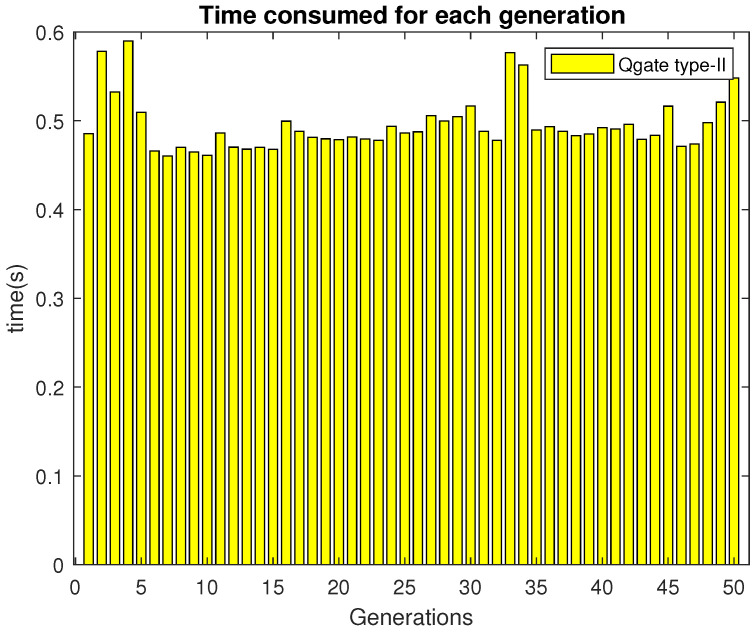
Time consumed per generation by 2D-QGA type-II.

Each drone is assigned to one task, and each task is allocated at least one drone. Therefore, the constraints are satisfied by the 2D-QGA type-II. The time consumed to execute each generation is shown in Figure 14.

It can be observed that most of the generation consumed around 0.48 s.

**Figure 15:**
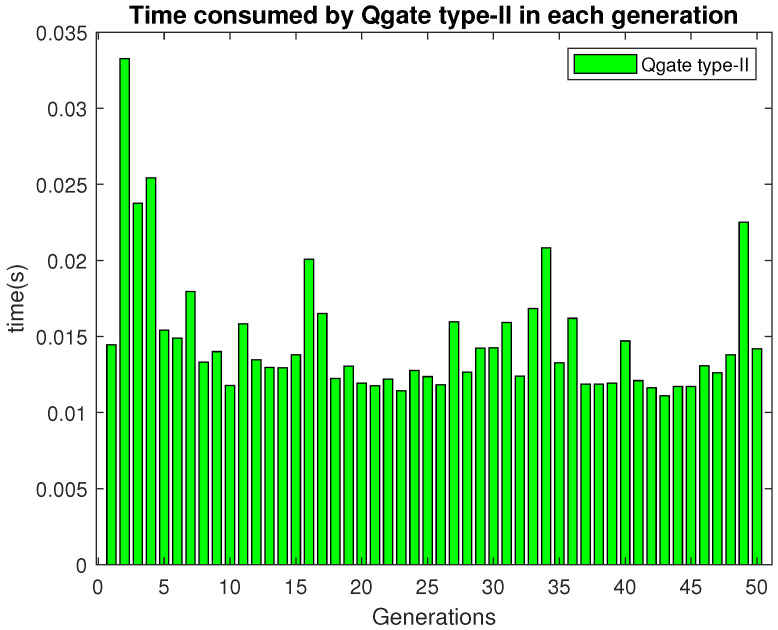
Time consumed by Qgate type-II in each generation.

The time to execute Qgate type-II in each generation is shown in Figure 15. In most of the generations, Qgate type-II needs around 0.011–0.014 s for execution.

The results generated using the proposed 2D-QGA algorithms (type-I and II) are discussed individually. However, the effect of quantum computation in 2D-QGA should be evaluated. The appropriate way should be to compare 2D-QGA with an algorithm which solves similar problems. It has been discussed in the introduction section that 2D-GA can solve the problems with two-dimensional representation. Therefore, the performance of 2D-QGA is compared to 2D-GA when they are used to solve the task allocation problem. The results obtained are discussed in the following section.

## 5. Comparison Study

The whole study is divided into three parts. In each part, two algorithms among 2D-GA, 2D-QGA type-I, and 2D-QGA type-II are compared. In the first part, the comparison between 2D-GA and 2D-QGA type-I is presented. In the second part, 2D-GA and 2D-QGA type-II are compared, and in the last part 2D-QGA, type-I and II are compared. The algorithms of each part are executed to solve the task allocation problem. It can be mentioned that the task and drone positions remain identical for each part. The comparison is performed by executing the pair of algorithms of each part for a finite number of iterations. In this study, the number of iterations considered to be 50. The general structure of the iteration is shown in Algorithm 8. In the algorithm, the iteration number *N* is set as 50, but the user can select a different number.
**Algorithm 8** Iteration for each part N←50 **for**
iteration=1 to *N*
**do**  Execute Algorithm 1 of each part  Execute Algorithm 2 of each part **end for**

In each iteration, the algorithms are allowed to evolve for fifty generations. The comparison between algorithms in each part is based on the points as follows.

1. Average time consumed (execution time) per generation by each algorithm of each pair. It is measured by
  $$\mathrm{Average}\;\mathrm{time}\;\mathrm{consumed}\;=\frac{\sum_{i=1}^{N}\left(\mathrm{Total}\;\mathrm{time}\;\mathrm{consumed}\;/\;\mathrm{Maximum}\;\mathrm{generation}\right)}{\mathrm{No}.\;\mathrm{of}\;\mathrm{iterations}(N)}$$
  This is an important measure because it gives quantitative feeling about how each generation is evolving for each algorithm.
2. The percentage of iteration each algorithm of a pair converges in fewer generations. This measure shows how many times (or %) in a specific number of iterations one algorithm converges in fewer iterations compared to another one.
3. The percentage of iteration one algorithm of a pair takes less time compared to the other one. This is the time consumed by each algorithm of a pair to execute the maximum number of generation in one iteration.
4. The percentage of iteration one algorithm of a pair produces less cost compared to the other one.

These measures are essential to understand the importance of the features that 2D-QGA has. The results obtained for each part is discussed in the following sections.

### 5.1. Part 1: Comparison between 2D-GA and 2D-QGA Type-I

**Figure 16:**
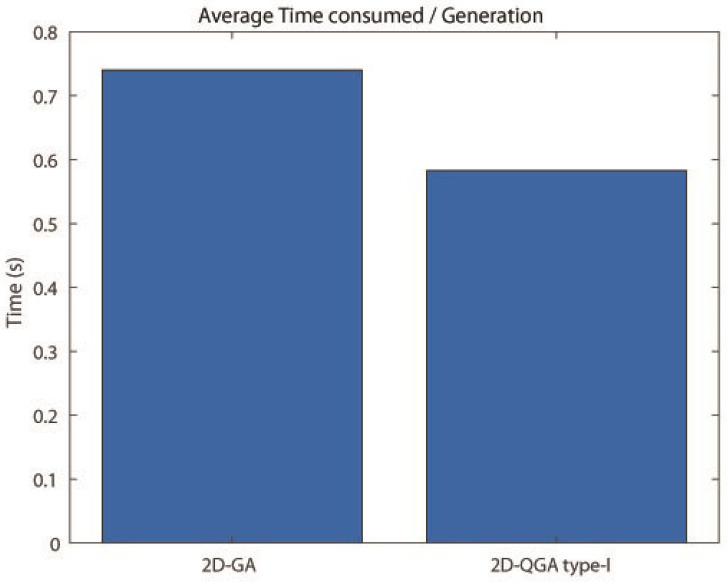
Average time consumed by each algorithms per generation compared to other.

**Figure 17:**
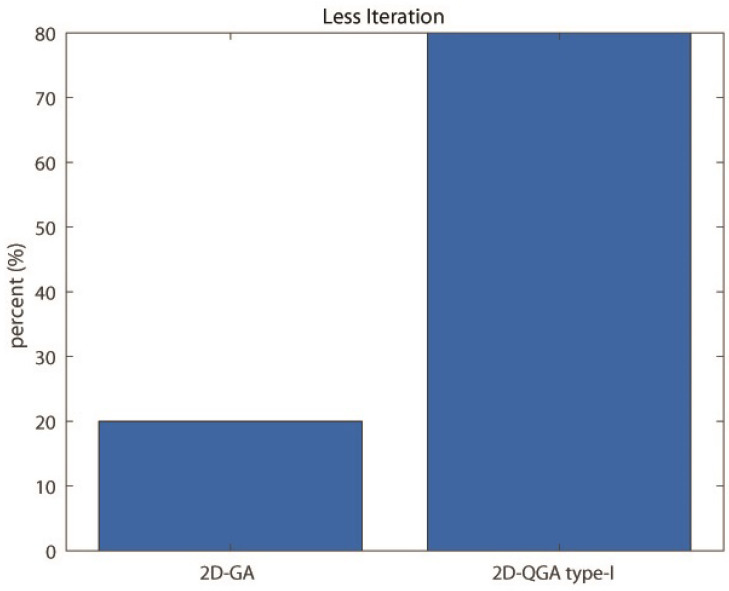
Percent of iterations each algorithm converges in less generations compared to other.

The comparison between 2D-GA and 2D-QGA type-I is given in this section. The population size of both 2D-GA and 2D-QGA type-I is considered to be 2000. The first comparison is about ‘Average time consumed’ by these algorithms. The comparison result is shown in Figure 16. It can be observed that 2D-GA consumed more time to execute each generation compared to 2D-QGA type-I. The operations involved in each generation for 2D-QGA type-I are designed by quantum computation. The next comparison is about the percentage of iteration the algorithms take fewer generations for convergence. The comparison is shown in Figure 17. It can be observed that 80% of iterations the 2D-QGA type-I takes fewer generations to converge.

**Figure 18:**
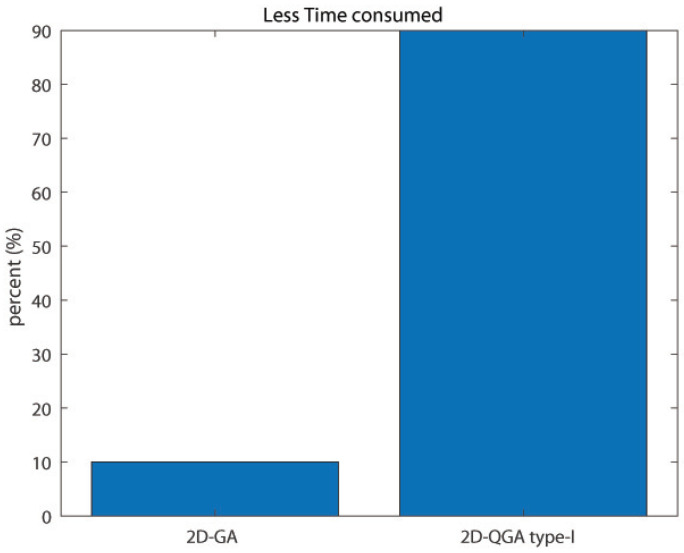
Percent of iterations each algorithm consumes less execution time compared to other.

**Figure 19:**
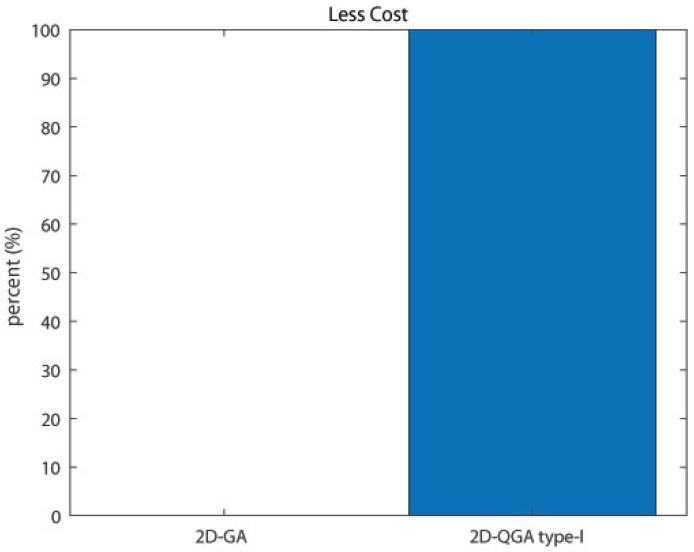
Percent of iterations each algorithm produces less cost compared to other.

Another important comparison is the total time taken by the algorithms to execute an equal number of generations. The result obtained is shown in Figure 18. It can be noticed that 2D-QGA type-I need lees execution time for all the iterations. The last and one of the most important comparison is the percentage of iterations the algorithms produce less cost. 2D-QGA type-I produces less cost for 100% of the iterations, as shown in Figure 19. The comparison is summarized in Table 2.

In this study, it is clear that 2D-QGA type-I is more efficient compared to 2D-GA. In the following section, the comparison between 2D-GA and 2D-QGA type-II is presented.

### 5.2. Part 2: Comparison between 2D-GA and 2D-QGA Type-II

**Figure 20:**
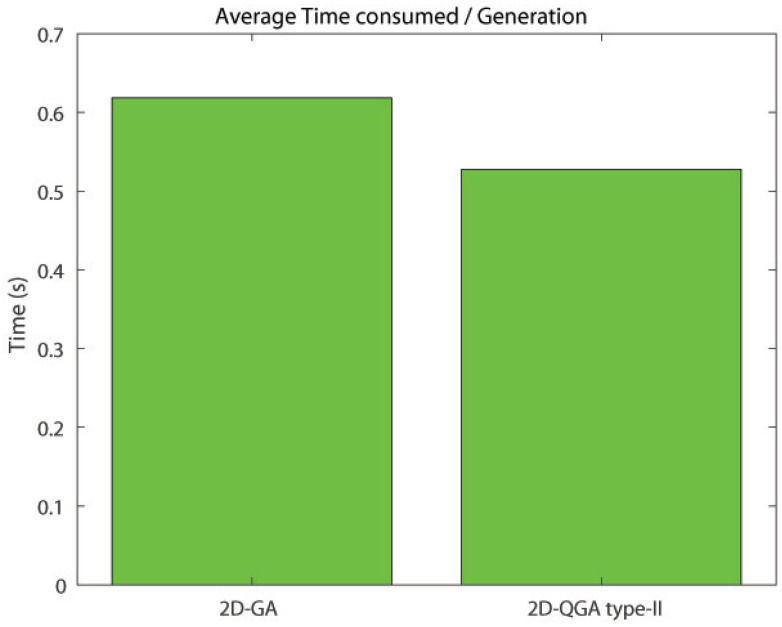
Average time consumed by each algorithms per generation compared to other.

**Figure 21:**
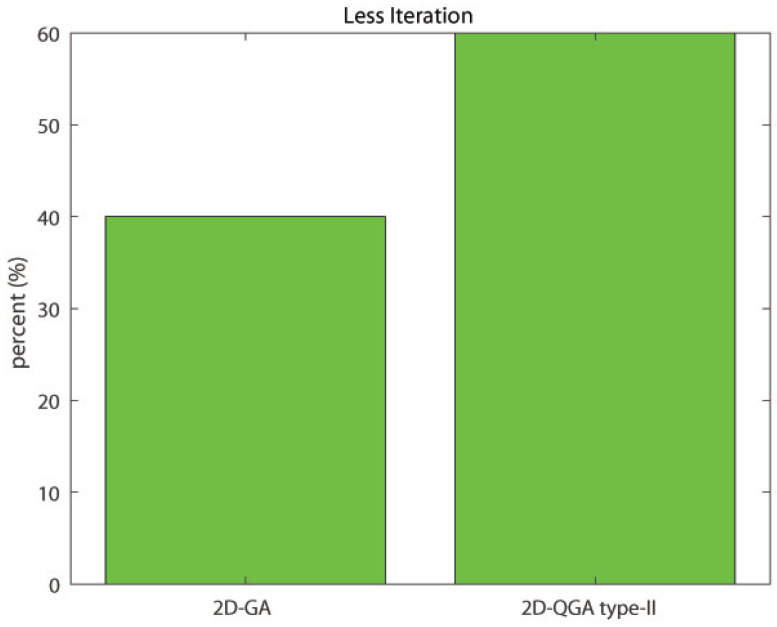
Percent of iterations each algorithm converges in less generations compared to other.

The comparison between 2D-GA and 2D-QGA type-II is given in this section. The population size of both 2D-GA and 2D-QGA type-II are 2000. The average time consumed by 2D-GA and 2D-QGA type-II is shown in Figure 20. The time consumed to execute each generation by 2D-QGA type-II is less compared to 2D-GA. Figure 21 shows the percentage of iterations the algorithms converge with fewer generations. It is clear that 2D-QGA type-II converges with fewer generations for 60% of iterations.

**Figure 22:**
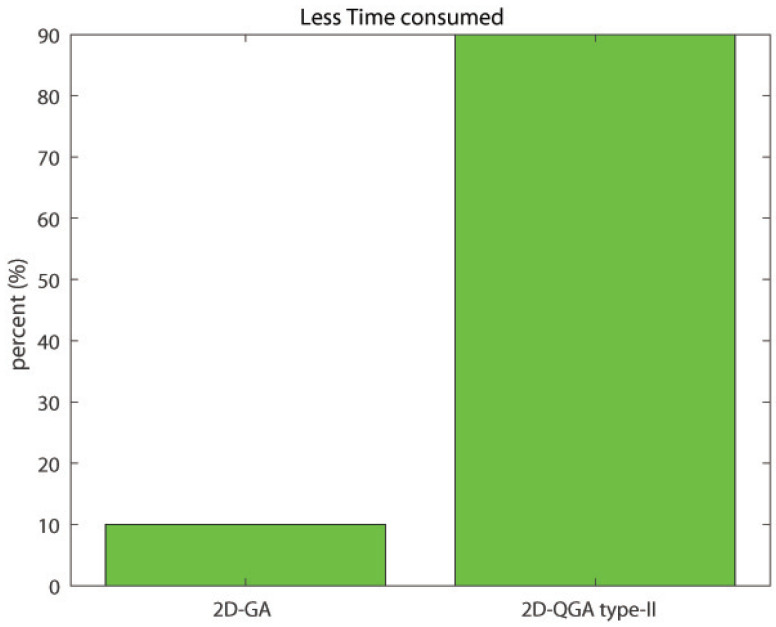
Percent of iterations each algorithm consumes less execution time compared to other.

The total time of execution of the same number of generations is compared in Figure 22. 2D-QGA type-II has consumed less execution time for 90% of iterations.

**Figure 23:**
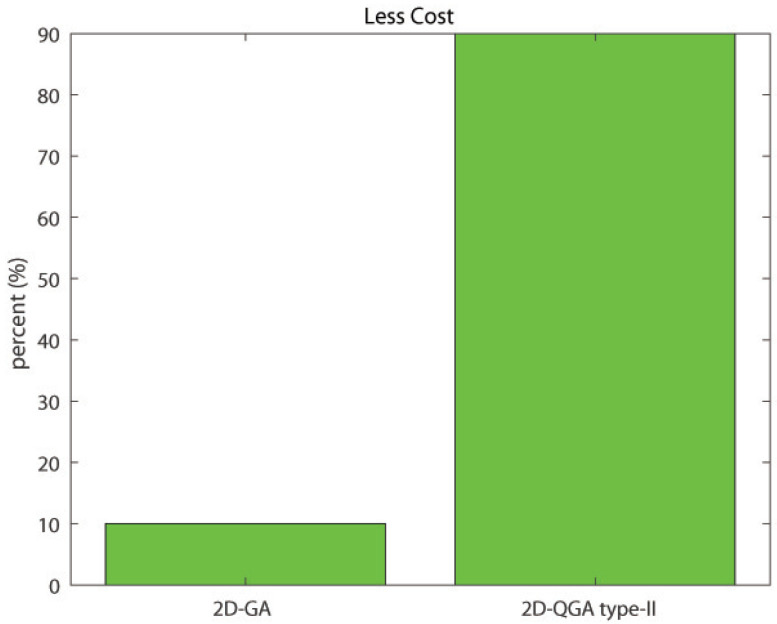
Percent of iterations each algorithm produces less cost compared to other.

The comparison of percent of less cost generation is shown in Figure 23. It can be noticed that 2D-QGA type-II produces less cost for 90% of iterations. The comparison is summarized in Table 3.

The comparison between 2D-GA and 2D-QGA (type-I and II) is made in parts 1 and 2. Both types of 2D-QGA have performed much better compared to 2D-GA in terms of average execution time per generation, per cent of iterations each algorithm converges in fewer generations, consumes less execution time, and produces less cost. Moreover, the quantum computation helped the 2D-QGA to reduce the number of generations for convergence and overall execution time. Therefore the 2D-QGA speeds up the computation for the class of problems discussed in this paper.

It is clear that both types (I and II) of 2D-QGA are more efficient than 2D-GA. Also, there should be a similar comparison between them to identify the more efficient one. This comparison primarily reflects the influence of the Qgate operation involved in each type of 2D-QGA. In part 3, the comparison between 2D-QGA type-I and type-II is presented.

### 5.3. Part 3: Comparison between 2D-QGA Type-I and 2D-QGA Type-II

The comparison between 2D-QGA type-I and 2D-QGA type-II is given in this section. For a proper comparison, the population size of both 2D-QGA type-I and 2D-QGA type-II is considered to be 2000.

**Figure 24:**
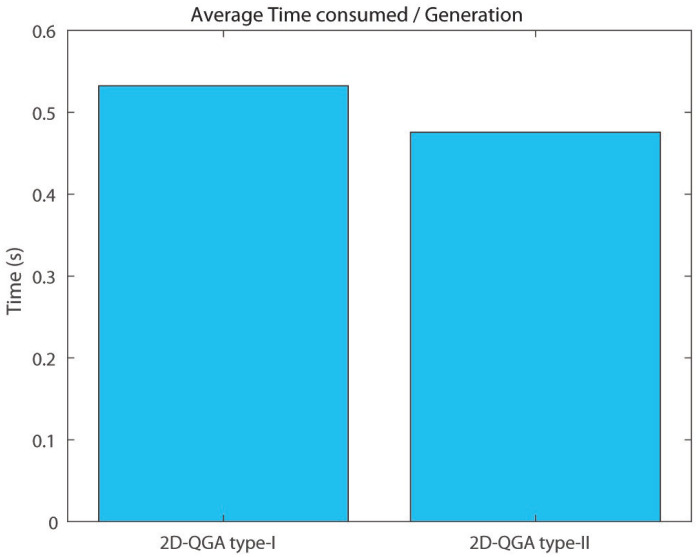
Average time consumed by each algorithms per generation compared to other.

**Figure 25:**
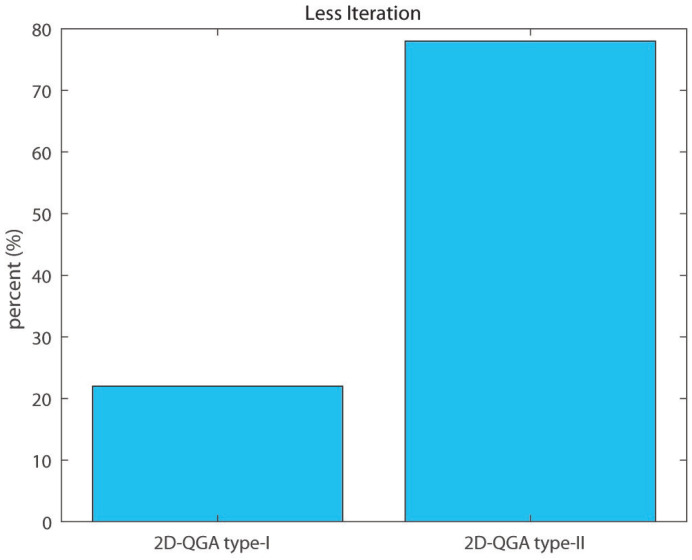
Percent of iterations each algorithm converges in less generations compared to other.

**Figure 26:**
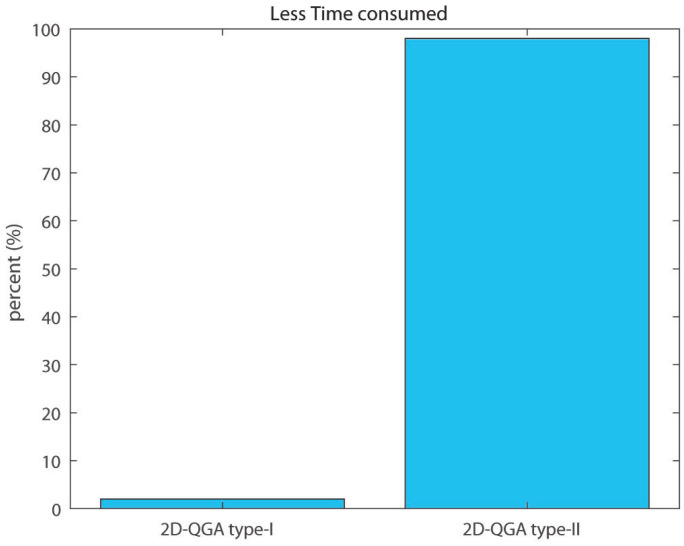
Percent of iterations each algorithm consumes less execution time compared to other.

It can be observed that the average execution time per generation consumed by 2D-QGA type-II (around 0.47 s) is less than 2D-QGA type-I (around 0.55 s) as shown in Figure 24. The percentage of iteration each algorithm converges in fewer generations is shown in Figure 25. It can be observed that 78% of the iterations, 2D-QGA type-II converges in fewer generations compared to 2D-QGA type-I. In case of total execution time, 2D-QGA type-II consumes less for almost all of the iterations (97%) as shown in Figure 26.

**Figure 27:**
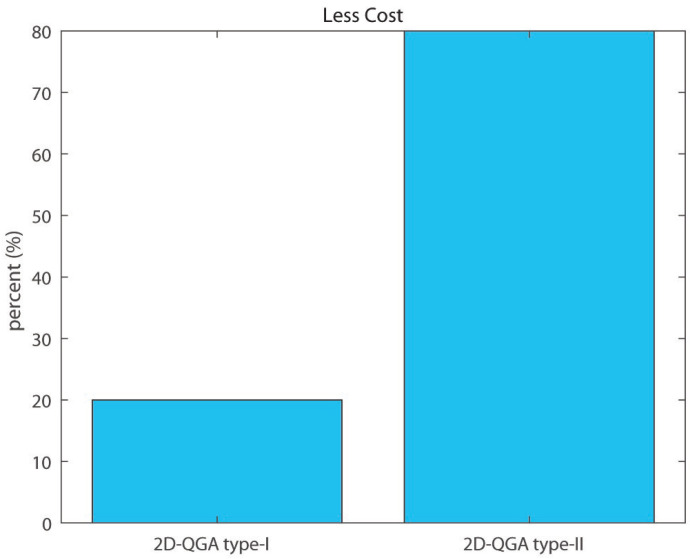
Percent of iterations each algorithm produces less cost compared to other.

Percent of iterations each algorithm produces less cost is shown in Figure 27. 2D-QGA type-II produces less cost for 80% of the iterations. The comparison is summarized in Table 4.

**Figure 28:**
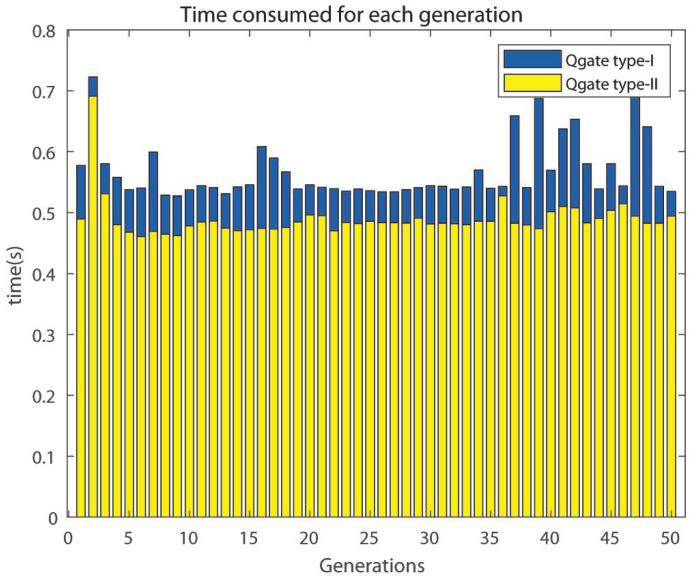
Time consumed by 2D-QGA type-I and II for all generation.

In addition to the comparison shown above, it is important to compare the execution time of 2D-QGA type-I and II for each generation in the same iteration. This comparison is shown in Figure 28. The execution time for each generation for type-I is more than type-II, i.e., type-II is faster. It can be observed that type-II takes around 0.1 s less time per generation compared to type-I and saves around 4–5 s over 50 generations. The saving of time increases if the number of generations is increased. The main reason behind type-II being faster is the time consumed by the Qgate process in type-II is much less than type-I.

**Figure 29:**
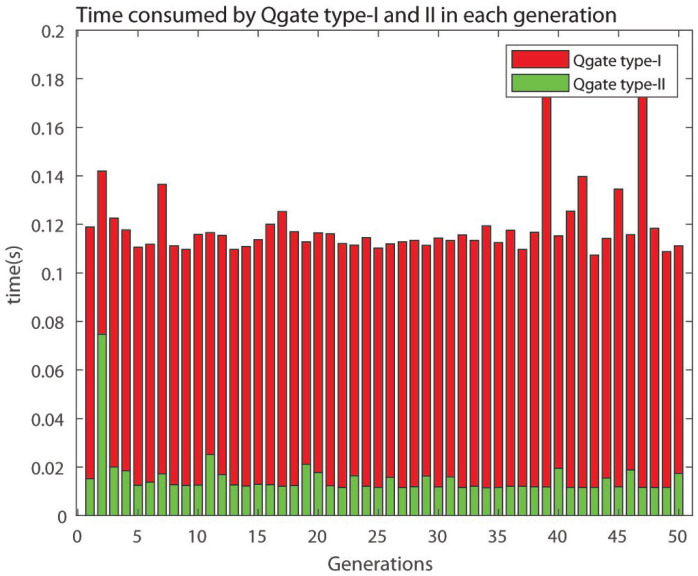
Time consumed by Qgate type-I and II in each generation.

The time consumed by Qgate of type-I and II is shown in Figure 29. The time consumed by Qgate type-I and II is around (average value) 0.12 s and 0.017 s respectively.

It is clear that 2D-QGA type-II has the advantage over type-I in terms of execution time per generation, total execution time, and convergence iterations. These advantages are important features of 2D-QGA type-II, which are useful for solving problems having two-dimensional representation. There are a few factors that can affect the performance of the proposed algorithm. The quality of optimal solution depends on the population size, the number of generations, formulation of appropriate cost function etc.

## 6. Conclusions

The simulation study showed that both types of 2D-QGA (type-I and II) had consumed less average execution time per generation compared to 2D-GA. This leads to less total execution time for 2D-QGA. Almost all the time (80–90%) the 2D-QGA produces less cost consuming fewer generations compared to 2D-GA. All of these improvements are achieved with population size much less than 2D-GA. The proposed Qgate type-I and II speeds up the computation. In particular, the type-II is faster between the two types. Therefore the 2D-QGA is a potential algorithm for solving problems with two-dimensional representation.

## Figures and Tables

**Table 1:** Look-up table for $\theta_{i}$.

$r_{i}$	$b_{i}$	f(r)<f(b)	$\Delta \theta_{i}\,\pi$	$s(\alpha_{i},\beta_{i})$			
				$\alpha_{i}\beta_{i}>0$	$\alpha_{i}\beta_{i}<0$	$\alpha_{i}=0$	$\beta_{i}=0$
0	0	false	0.2π	0	0	0	0
0	0	true	0	0	0	0	0
0	1	false	0.5π	0	0	0	0
0	1	true	0	−1	+1	±1	0
1	0	false	0.5π	−1	+1	±1	0
1	0	true	0	+1	−1	0	±1
1	1	false	0.2π	+1	−1	0	±1
1	1	true	0	+1	−1	0	±1

**Table 2:** Comparison of 50 iterations: Part 1.

	2D-GA	2D-QGA Type-I
Avg. time/gen	0.74 s	0.58 s
Less gen.	20%	80%
Less exe. time	10%	90%
Less cost	0%	100%

**Table 3:** Comparison of 50 iterations: Part 2.

	2D-GA	2D-QGA Type-II
Avg. time/gen	0.62 s	0.52 s
Less gen.	40%	60%
Less exe. time	10%	90%
Less cost	10%	90%

**Table 4:** Comparison of 50 iterations: Part 3.

	2D-QGA Type-I	2D-QGA Type-II
Avg. time/gen	0.55 s	0.47 s
Less gen.	22%	78%
Less exe. time	3%	97%
Less cost	20%	80%

## Data Availability

Not applicable.
